# An Assessment of the Spatial and Temporal Variability of Biological Responses to Municipal Wastewater Effluent in Rainbow Darter (*Etheostoma caeruleum*) Collected along an Urban Gradient

**DOI:** 10.1371/journal.pone.0164879

**Published:** 2016-10-24

**Authors:** Meghan L. M. Fuzzen, Leslie M. Bragg, Gerald R. Tetreault, Paulina A. Bahamonde, Rajiv N. Tanna, Charles J. Bennett, Mark E. McMaster, Mark R. Servos

**Affiliations:** 1 Department of Biology, University of Waterloo, Waterloo, Ontario, Canada; 2 Water Science Technology Directorate, Environment and Climate Change Canada, Canada Center for Inland Waters, Burlington, Ontario, Canada; 3 Canadian Rivers Institute and Department of Biology, University of New Brunswick, Saint John, New Brunswick, Canada; Northwest Fisheries Science Center, UNITED STATES

## Abstract

Municipal wastewater effluent (MWWE) and its constituents, such as chemicals of emerging concern, pose a potential threat to the sustainability of fish populations by disrupting key endocrine functions in aquatic organisms. While studies have demonstrated changes in biological markers of exposure of aquatic organisms to groups of chemicals of emerging concern, the variability of these markers over time has not been sufficiently described in wild fish species. The aim of this study was to assess the spatial and temporal variability of biological markers in response to MWWE exposure and to test the consistency of these responses between seasons and among years. Rainbow darter (*Etheostoma caeruleum*) were collected in spring and fall seasons over a 5-year period in the Grand River, Ontario, Canada. In addition to surface water chemistry (nutrients and selected pharmaceuticals), measures were taken across levels of biological organization in rainbow darter. The measurements of hormone production, gonad development, and intersex severity were temporally consistent and suggested impaired reproduction in male fish collected downstream of MWWE outfalls. In contrast, ovarian development and hormone production in females appeared to be influenced more by urbanization than MWWE. Measures of gene expression and somatic indices were highly variable between sites and years, respectively, and were inconclusive in terms of the impacts of MWWE overall. Robust biomonitoring programs must consider these factors in both the design and interpretation of results, especially when spatial and temporal sampling of biological endpoints is limited. Assessing the effects of contaminants and other stressors on fish in watersheds would be greatly enhanced by an approach that considers natural variability in the endpoints being measured.

## Introduction

Municipal wastewater effluent (MWWE) is the largest (by volume) source of pollution in the aquatic environment in Canada [1]. Over the past century, municipal wastewater treatment plants (MWWTPs) have enhanced their processes to effectively remove pathogens, organic matter, and some portion of the nitrogenous wastes from the effluents. The use of these treatment technologies has vastly improved public and environmental health [2, 3]. While progress in treatment processes has greatly reduced their environmental impacts, new challenges have arisen. Chemicals of emerging concern (CECs), such as human steroid hormones, and pharmaceuticals and personal care products, are passed through the body or washed down sink drains and enter municipal wastewater systems [4, 5]. MWWTPs are not currently designed to remove the diversity of CECs that enter the wastewater streams [6]. Although some compounds are fully degraded through existing wastewater treatment processes, others are either transformed into other compounds or remain intact and released into the receiving environment [7–9]. Thus, numerous CECs have been detected in surface waters around the world [10].

The presence of CECs in aquatic environments is of concern because of their wide distribution and their potential to have adverse effects on aquatic organisms [11, 12]. The concentrations of these compounds in surface waters are typically very low, often below the detection limits of current analytical methods [13]. However, it has been demonstrated that CECs can impair the endocrine function of aquatic organisms at environmentally relevant concentrations [14]. This high activity at low concentrations is due to the similarity of the structure of these chemicals to endocrine hormones, allowing them to bind to endocrine receptors [15]. Estrogenic compounds such as 17α-ethinylestradiol (EE2), which are ubiquitous in MWWE [10], can impair reproduction in fish populations at concentrations of a few ng/L [16–18]. A study in which a whole-lake was dosed with an average of 5 ng/L of EE2 for 3 consecutive years found that fathead minnows (*Pimephales promelas*) exhibited disrupted reproductive health followed by a collapse of the population [19, 20].

Despite the strong relationship between exposure to an estrogenic compound and the collapse of a population of fish in the whole-lake study, it remains difficult to make direct associations between CECs in effluents and adverse affects in aquatic organisms. Laboratory [21–23] and caging [24–26] exposures of aquatic organisms to MWWE have improved our understanding of the changes in biomarkers that occur as a result of exposure to CECs [27]. Adverse outcome pathways (AOPs) have synthesized the knowledge gained in laboratory and caging studies by linking stressors to biological responses across levels of biological organization. AOPs identify molecular initiating events, key biological events, and adverse outcomes [28]. Although many AOPs are able to predict adverse outcomes to groups of stressors under controlled laboratory conditions, they have not yet expanded to include the complexities of exposure in aquatic environments.

There are many additional variables present in the field that complicate the assessment of the effects of CECs in MWWE. Wastewaters are complex mixtures of chemicals and nutrients that can vary temporally, and their composition is also dependent on the degree and type of treatment processes [7, 13, 29, 30]. Additionally, natural systems contain many other anthropogenic and natural stressors that can vary spatially and temporally across watersheds, creating confounding or cumulative effects [31, 32]. As a result, measures of fish health in receiving waters vary widely over time. The main challenge we face in assessing the potential adverse effects of CECs in MWWE is separating changes in biological indicators due to exposure from changes due to natural variability. The variability of biological measures associated with endocrine disruption has not been well explored in wild fish in the literature.

A number of studies have examined wild fish in areas influenced by MWWE outfalls to assess the effects associated with CECs [33–41]. While many studies have examined key biological responses associated with CECs in MWWE, few have examined the variability in these responses. The variability that has been examined includes variability due to species differences [42], spatial variation [36], and seasonal variation [43, 44]. To date, only 1 study has examined the annual variability of reproductive disruption [43]. Although consistent responses were found between 2 years of study in that report, there is still a great need for additional information concerning the amount of variability in the response of fish populations to MWWE and the consistency of the response over time. To be predictive, biological monitoring programs need to be able to confidently detect a change in a biological measure that can be associated with a source or stressor. Once a change is confidently detected and placed within the context of an adverse outcome pathway, more appropriate and effective management decisions can be made.

The aim of this study is to better understand the spatial and temporal variability of key biological measures associated with CEC exposures. A large data set was assembled by compiling previous research data and gathering additional samples from the Grand River, Ontario, which is an area with known MWWE impacts [45–47]. The intense study of this unique system presents an opportunity in which to address questions about the variability of biological responses exposed to MWWE.

## Materials and Methods

Rainbow darter (*Etheostoma caeruleum*) are a small-bodied, benthic-dwelling, sexually dimorphic percid species. Reproductive maturity is reach at 1 year of age and adults spawn annually (asynchronously in clutches) from mid-April to late May. This species was chosen as a model because it is highly abundant in the watershed and has low mobility. To assess reproductive health, fish were collected after gonad recrudescence in the fall (late October/early November) and just before the spawning season in the spring (late March/early April).

**Fig 1:**
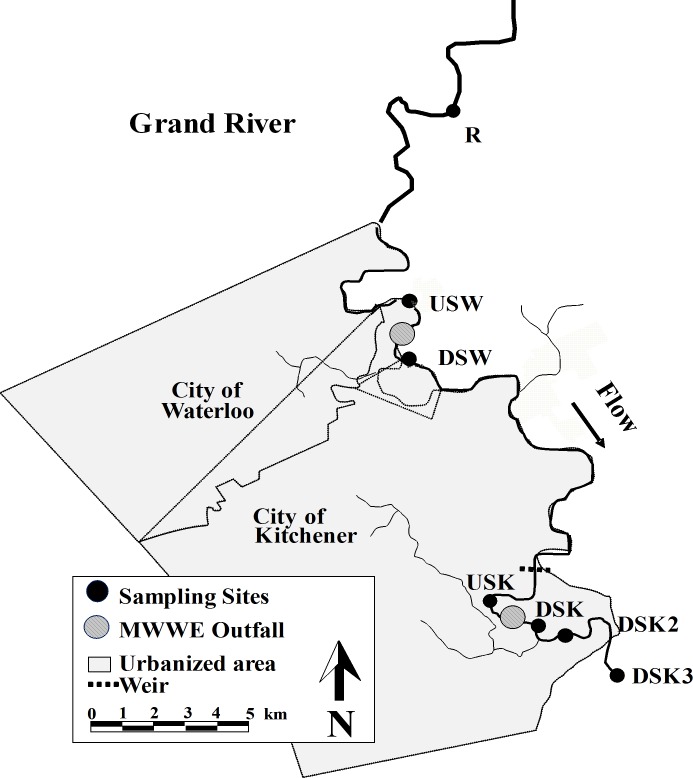
Map of sampling area in the Grand River watershed (southern Ontario, Canada). Fish and surface water were collected from 1 reference (R) site, 2 sites upstream (US), and 4 sites downstream (DS) of municipal wastewater effluent (MWWE) outfalls.

Surface water chemistry and biological measures were collected in fall and spring seasons for up to 5 years from 7 sites. These sites were dispersed through an urban gradient that encompasses the outfalls of 2 MWWTPs in the Grand River, Ontario, Canada (Fig 1). In addition to novel data collected in fall of 2011, spring of 2012, and fall of 2012, data were pooled from studies conducted in the Grand River watershed from fall 2007 to spring 2011. These studies used similar methodology to collect rainbow darter and measure indicators of their health. The data from fall 2007 to spring 2011 were originally used to address independent research questions related to the endocrine-disrupting effects of wastewater [45–50].

The Grand River watershed supports close to 1 million people spread across several population centers. There are 30 MWWTPs in the watershed, which vary greatly in terms of the size of population served and process type. The 2 largest treatment plants (Waterloo and Kitchener) are located close to one another in the most urbanized central reaches of the watershed. At the time of the studies, both treatment plants were operated with an activated sludge process with minimal or partial nitrification, as described in Table 1.

**Table 1:** Properties of Waterloo and Kitchener municipal wastewater treatment plants from 2007 to 2011 [51].

Municipal wastewater treatment plant	Population served	Treatment process	Capacity rating (m^3^/day)	Volume (m^3^/day)
Waterloo	120,265–127,688	Conventional activated sludge with chemical phosphorus removal, anaerobic sludge digestion, and chlorine disinfection	54,600	41,358–47,562
Kitchener	215,247–227,761	Conventional activated sludge with chemical phosphorus removal, anaerobic sludge digestion, sodium hypochlorite disinfection, and sodium bisulphite dechlorination	122,700	64,329–74,935

### Site description

Fish were collected across an urban gradient that included the outfalls of the 2 largest wastewater treatment plants (Fig 1). Although the number of sites varied among seasons and years because of changes in the objectives of the original collections, access, and weather conditions, a subset of sites were selected for analysis in this study. Up to 7 sites from each sampling period were included. A rural reference (R) site, an upstream and downstream site at the Waterloo MWWTP (USW, DSW), and an upstream site and 3 downstream sites at the Kitchener MWWTP (USK, DSK, DSK2, DSK3) were selected.

### Surface water and effluent sampling, preparation, and analysis

Surface water was collected from the fish collection sites during fall and spring of 2010, 2011, and 2012 and analyzed for selected pharmaceuticals and personal care products as well as nutrients (ammonia). For pharmaceutical analysis, grab samples of surface water were collected in triplicate (1 sample each collected from the near, center, and far bank of the river, or within the plume of effluent for sites downstream of MWWTPs) using pre-cleaned 500 mL amber glass bottles. Samples were preserved on site with sodium azide (1 g/L) and ascorbic acid (50 mg/L) and stored at 4°C until extraction. The preparation, extraction, and analyses of water samples are described in detail by Tanna *et al*. [52]. Briefly, compounds were analyzed using solid phase extraction followed by liquid chromatography and tandem mass spectrometry (LC-MS/MS) using an Agilent 1200 HPLC (Mississauga, ON, Canada) coupled to an Applied Biosystems 3200 QTRAP mass spectrometer (ABSciex, Concord, ON, Canada). Additional grab samples of surface water were collected using 500 mL plastic bottles and were submitted to Maxxam Analytics (Mississauga, ON, Canada) for analysis of ammonia.

### Field fish collections

Previous studies identified rainbow darter (*E*. *caeruleum*) as the most abundant fishes in riffle/run habitats throughout the watershed [53]. In this study, all animals were handled in strict accordance with the principles of the Canadian Council on Animal Care, and the protocol was approved by the Animal Care Committee of the University of Waterloo (Permit Numbers 04–24, 08–08, and 10–17). Fish were stunned using a backpack electrofisher (Smith-Root LR-24), collected with a net, and placed into aerated buckets. Fish were brought to an on-site sampling trailer where the length (to 0.1 cm), weight (to 0.001 g), gonad weight (to 0.001 g), and liver weight (to 0.001 g) were measured. Livers were placed into cryo-vials and flash frozen in liquid nitrogen for gene expression analysis. Gonads were divided and alternately allocated to gene expression, steroid, or histological analysis. The number of fish collected and the endpoints studied varied in some years, depending on the research questions being addressed. A table showing the sample size for each endpoint in all seasons/years is provided in Table A in S1 File.

### Quantification of mRNA by real-time reverse transcriptase-polymerase chain reaction

RNA extraction, cDNA synthesis, and real-time reverse transcriptase-polymerase chain reaction (qPCR) were performed as described by Bahamonde *et al*. [50]. The RNA integrity of fall 2010 samples was reported by Bahamonde *et al*. [50]. The RNA integrity of fall 2011 and 2012 samples was evaluated using A260/A280 and the Tape Station (2200, Agilent Technologies) with the RNA Screen Tape according to the manufacturer’s protocol. RNA Integrity Number equivalent (RIN^e^) values averaged 8.93 ± 0.07 (mean ± SE). Briefly, 1 μg total RNA was reverse transcribed to cDNA using iScript cDNA reverse transcriptase (Bio-Rad Laboratories, Mississauga, ON, Canada) according to the manufacturer’s protocols. Gene-specific primer sequences for rainbow darter, vitellogenin (*vtg*), 18S ribosomal RNA (*18s*), elongation factor 1-α (*ef1α*), and *β-actin* were obtained from Bahamonde *et al*. [50] and are listed in Table 2. Reference genes were combined using geNORM [54], and normalized expression levels for target genes (*vtg*) were extracted using CFX Manager 2.1 software (Bio-Rad Laboratories) using a relative ΔΔCq method. Expression data are reported as fold change ± SE from reference (R) male mRNA levels.

**Table 2:** Real-time PCR primers used to measure mRNA levels in rainbow darter livers. R^2^ and efficiency (E) were calculated from fall 2011 and fall 2012 samples only.

Gene	Forward primer (5’-3’)	Reverse primer (5’-3’)	Amplicon size (bp)	R^2^	E (%)
*18s*	CGGTTCTATTTTGTGGGTTTTC	ACCTCCGACTTTCGTTCTTG	172	0.992	105.5
*ef1α*	ATCGGCGGTATTGGAACTG	CGGATTTCTTTGACGGACAC	197	0.997	102.9
*β-actin*	CCAACAGGGAGAAGATGACAC	GAGGATGGCGTGAGGTAGAG	188	0.993	112.5
*vtg*	CTACGCTCATTCCTGGGTTC	CAGTGGCAGTCGTTGTCCT	188	0.996	99.0

### In vitro steroid production

Gonad *in vitro* sex steroid production was determined as described in detail by Fuzzen *et al*. [45]. Briefly, gonads collected in the field were transported on ice to the laboratory, where up to 20 mg of tissue was placed into a 24-well cell culture plate with 1 mL of media 199 (Sigma-Aldrich) and stimulant and incubated at 16°C for 24 h. Stimulation of sex steroid production differed between years. From fall 2007 to spring 2011, Forskolin was used (as described by Tetreault *et al*. [47]). From fall 2011 onward, steroid production was stimulated with 10 IU of human chorionic gonadotropin (hCG) solubilized in media 199. At the end of the incubation period, media was removed from the wells, placed into centrifuge tubes, and stored at -80°C for analysis of sex steroid hormones. Testosterone (T) and 11-ketotestosterone (11KT) from testis incubations and 17β-estradiol (E2) and T from ovary incubations were measured by radioimmunoassay (RIA) as described by McMaster *et al*. [55] from fall 2007 to fall 2011 and by enzyme immunoassay (EIA) kit (Cayman Chemicals, Ann Arbor, MI, USA) according to manufacturer’s directions in spring and fall 2012. Briefly, 200 μL (RIA) or 50 μL (EIA) aliquots of each sample were measured in duplicate.

### Histological processing and analysis

One lobe of gonad tissue was placed into an individual histocassette in Davidson’s solution for histological analysis and fixed for 48 h. Samples were processed and slides made as described by Fuzzen *et al*. [45]. Males were analyzed for the presence and severity of intersex. Intersex condition is classified as the presence of ovarian tissue (oocytes) in predominantly male gonads. Males were given an intersex severity score from 0 to 7. The scoring system for determining intersex severity in male rainbow darter was based on number of oocytes, oocyte development, and proportion of ovarian tissue versus testicular tissue. The rationale for the scoring system is described in detail by Bahamonde *et al*. [46] and Fuzzen *et al*. [45]. In addition to intersex, males were assessed for the relative proportion of spermatogonia, spermatocytes, spermatids, and spermatozoa as described by Tetreault *et al*. [47]. For each female, the relative proportion of perinuclear, cortical alveolar, pre-vitellogenic, and mature (mid-vitellogenic, late-vitellogenic, and vitellogenic) oocytes was determined for all females collected, as described previously [45–47].

### Somatic indices

Gonad somatic index (GSI; gonad weight / total body weight *100), liver somatic index (LSI; liver weight / total body weight *100), and condition factor (K; weight/ (length^3^) *100) were determined for all individuals collected and the mean was determined for males and females at each site in each season and year.

### Statistical analyses

Data from the 3 new field seasons and from the earlier studies conducted from fall 2007 to spring 2011were compiled into a single database. The mean, standard deviation, and standard error were determined for each endpoint within each season, year, site, and sex (for biological data). For chemistry data, a 2-way analysis of variance (ANOVA) was conducted to test for the effects of site, year, and interactions between site and year. A Tukey’s post-hoc test was conducted to test for differences between individual sites. This test was performed on data for ammonia as well as for pooled CECs (consisting of naproxen, ibuprofen, venlafaxine, carbamazepine, and triclosan). For chemical profiles of select pharmaceuticals a metric to test for statistical differences was placed on each graph. For all biological data, the same metric to test for statistical differences and a second metric to test for biological differences were placed onto the graphs. Both of these metrics were calculated from a pooled data set from the R site in all years for each season and sex. The first metric calculated was the 95% confidence interval of the pooled data from the R site determined using SPSS (Version 23; IBM, New York, USA). Values that exceeded the 95% confidence interval of the mean R site value were considered to be statistically different. The second metric calculated was the critical effect size (CES), which was based on thresholds set in the environmental effects monitoring program for pulp and paper from Environment Canada and recommendations from Munkittrick *et al*. [56]. The CES was set to ± 10% of the mean for the condition factor and ± 25% of the mean for all other measures. Values that exceeded the CES were considered to be biologically different.

### Cluster analyses

To further explore the data, we conducted 3 ordination tests to assess the influence of MWWE on rainbow darter biology. The first test was a principal coordinates analysis (PCO) ordination test, which was used to explore site clustering and test for seasonal effects. To determine which variables were responsible for the separation along the axis, a canonical analysis of principal coordinates (CAP) ordination was conducted. The third test, distance-based redundancy analysis (DISTLM), was used to explore what amount of the variation in biological variables changing at downstream sites was attributable to MWWE constituents. Ordination analyses were conducted on the mean of biological variables. Analyses were conducted on biological and chemical data normalized within the Primer+PERMANOVA software package (PRIMER-E Ltd) [57].

### Variability assessment of biological variables

The variability of several measures in the study was determined by normalizing each data point to the mean of its site. To test the variability of measures across levels of biological organization, 4 key endpoints in male rainbow darter (*vtg* expression, testicular testosterone production, spermatozoa proportion, and gonad somatic index) were compared at the R site in the fall season. Additionally, site-specific variability was assessed by comparing these same endpoints between the R site and the DSK site.

## Results

### Chemistry

**Fig 2:**
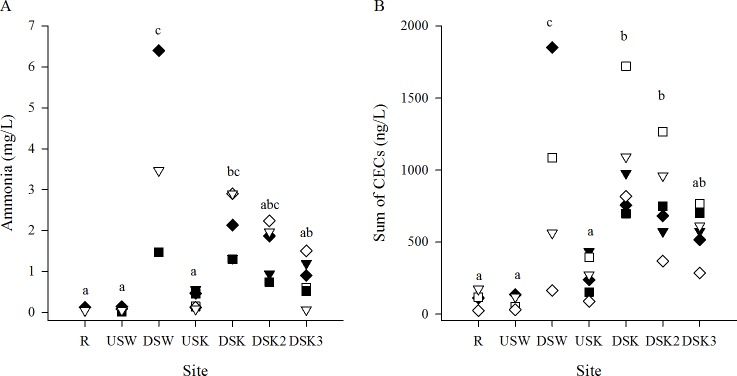
Concentration of nutrients and pharmaceuticals increases downstream of MWWTPs. (A) Mean concentration of ammonia (mg/L) and (B) the sum of dominant chemicals of emerging concern (naproxen, ibuprofen, venlafaxine, carbamazepine, and triclosan) in river surface water. Samples were collected in the fall (dark symbols) and in the spring (open symbols) of 2010 (squares), 2011 (diamonds), and 2012 (down-facing triangles). Sites that share a common letter are not significantly different (*p* > 0.05) as determined by a 2-way ANOVA with a Tukey’s post-hoc test (no interactions were found for season and site).

**Fig 3:**
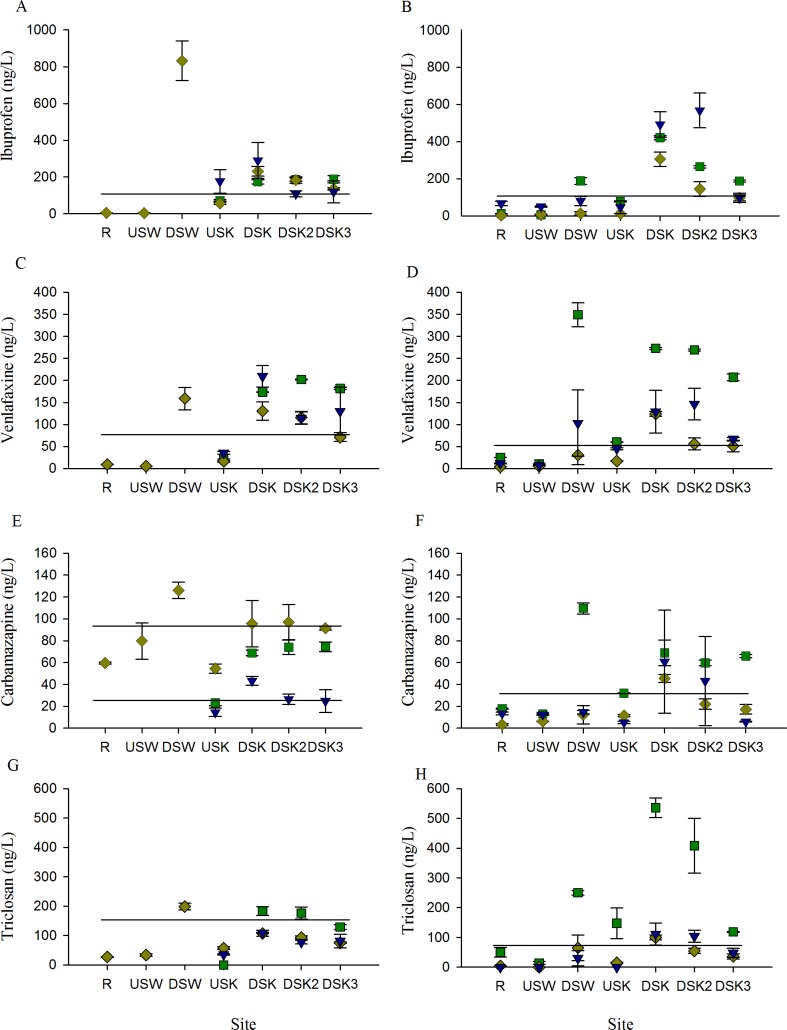
Profile of pharmaceuticals and personal care products in surface water. Mean concentration (± SE) of select pharmaceuticals and personal care products in river surface water through an urban gradient. Samples were collected in the (A, C, E, G) fall and in the (B, D, F, H) spring 2010 (green squares), 2011 (yellow diamonds), and 2012 (blue down-facing triangles). The solid lines indicate the 95% confidence interval calculated from the mean of the data from the rural reference site (R).

Surface water concentration of nutrients (ammonia in this study) and CECs (pharmaceuticals) increased downstream of both the Waterloo and Kitchener MWWTPs in all years and all seasons (Fig 2). The absolute concentration of nutrients and pharmaceuticals varied between seasons and years but the profiles remained consistent. The patterns of ammonia and pharmaceutical concentrations resembled one another, and when a regression analysis was conducted they were found to be directly correlated (R^2^ = 0.644, *p* < 0.05). The profiles of 4 of the 5 CECs investigated in this study (ibuprofen, venlafaxine, carbamazepine, and triclosan) that were found at high concentrations in surface water are presented in Fig 3. The detection of elevated levels of these compounds at sites downstream of MWWTPs demonstrates that MWWE is a source of measurable concentrations of pharmaceuticals in surface river water. These profiles also demonstrate that CECs persist in the watershed even after dilution due to mixing, because concentrations were higher at the DS3 site than at the R site (Fig 3).

### Gene expression

**Fig 4:**
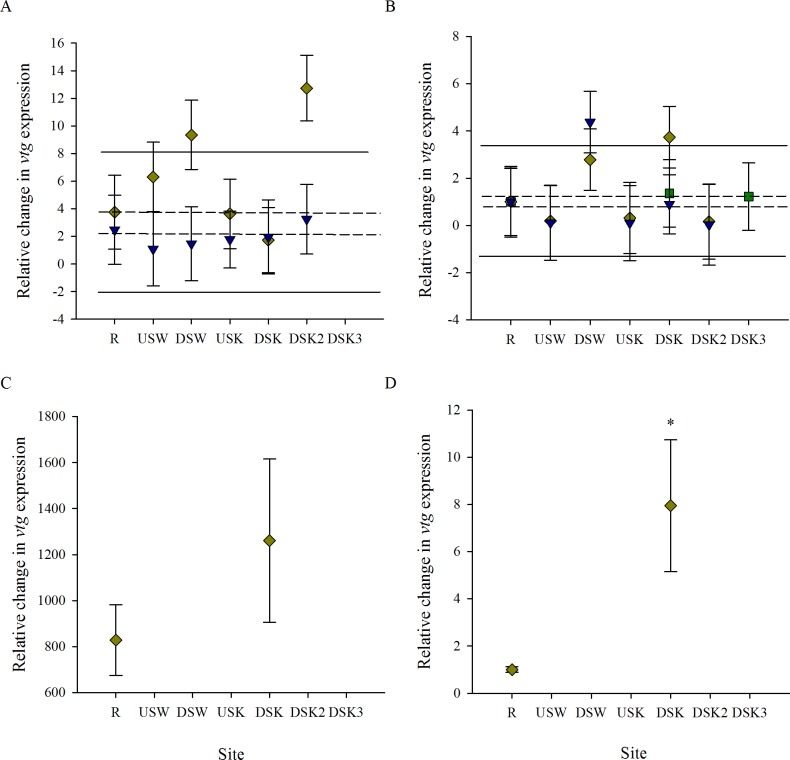
Vitellogenin (vtg) expression is higher in males collected downstream of MWWTP outfalls. Relative change in vitellogenin (*vtg*) expression in liver tissues of (A, C) female and (B, D) male rainbow darter collected from sites through an urban gradient, compared with that in R males. Expression was measured in the (A, B) fall of 2010 (green squares), 2011 (yellow diamonds), and 2012 (blue down-facing triangles), as well as in the (C, D) spring of 2011. The dashed line indicates the upper 25% critical effect size calculated from the mean of the data from the rural reference site (R) over all 3 years, and the solid line indicates the upper 95% confidence interval from the pooled R. In the spring (panels C, D), * indicates significant (*p* < 0.05) difference between R and DSK sites as determined by a Student’s t-test.

Quantitative measurement of female liver *vtg* in the fall and spring revealed no impact of MWWTP outfall on expression (Fig 4A and 4C). Although some increases in *vtg* were observed within the urban area in fall 2011, no additional increases were observed directly downstream of the MWWTP (Fig 4A). Male liver *vtg* was found to increase downstream of both MWWTP outfalls in some fall seasons and 1 spring season (Fig 4B and 4D).

### Steroids

**Fig 5:**
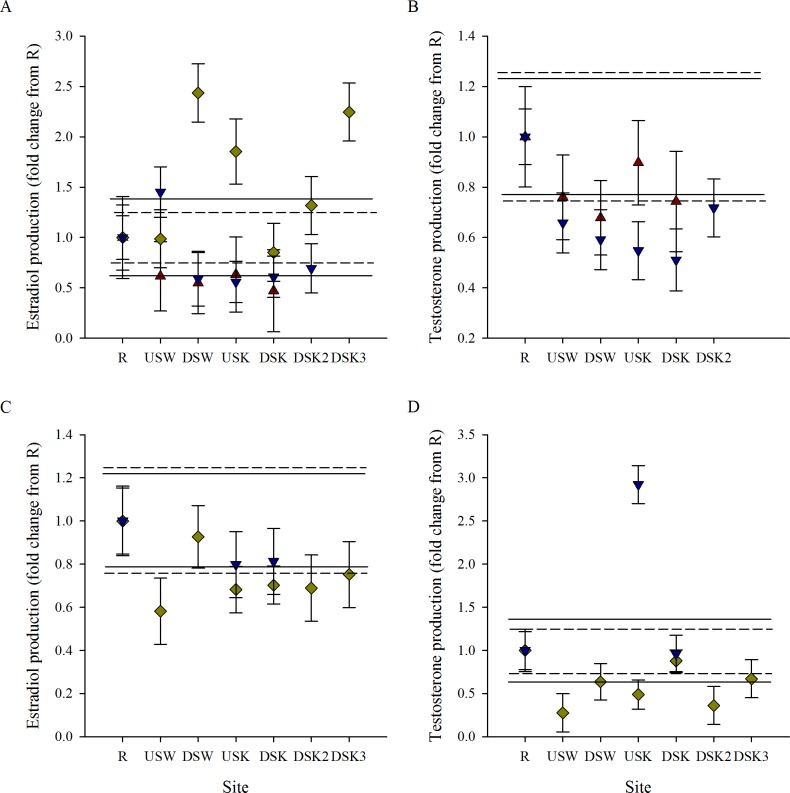
Hormone production of ovaries varies through the urban gradient and between seasons. Normalized (to mean R site) *in vitro* steroid production of (A, C) estradiol and (B, D) testosterone in stimulated ovarian tissues collected from rainbow darter in the wild through an urban gradient. Measurements were taken in the (A, B) fall of 2007 (red triangles), 2011 (yellow diamonds), and 2012 (blue down-facing triangles), and well as in the (C, D) spring of 2011 (yellow diamonds) and 2012 (blue down-facing triangles). The dashed line indicates the upper 25% critical effect size calculated from the mean of the data from the rural reference site (R), and the solid line indicates the upper 95% confidence interval from the pooled R.

**Fig 6:**
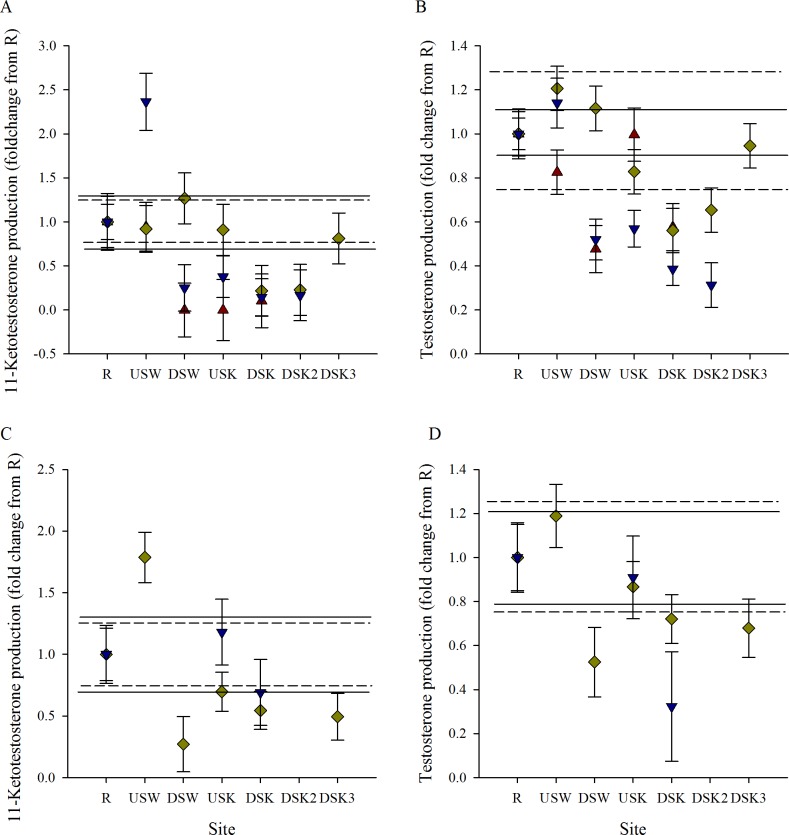
Hormone production of testes is reduced in male exposed to MWWE. Normalized (to mean R site) *in vitro* steroid production of (A, C) 11-ketotestosterone and (B, D) testosterone in stimulated testis tissue collected from rainbow darter in the wild through an urban gradient. Measurements were taken in the (A, B) fall of 2007 (red triangles), 2011 (yellow diamonds), and 2012 (blue down-facing triangles), as well as in the (C, D) spring of 2011 (yellow diamonds), and 2012 (blue down-facing triangles). The dashed line indicates the upper 25% critical effect size calculated from the mean of the data from the rural reference site (R), and the solid line indicates the upper 95% confidence interval from the pooled R.

*In vitro* gonad steroid production was measured in male and female rainbow darter in 3 fall field seasons and 2 spring field seasons. To compare the response among years, data were normalized to the reference site (R) within each field season. While trends appeared similar across years, absolute values of hormone measurement among years varied greatly (Table B in S1 File). Differences in absolute measures have also been noted in an inter-laboratory study of hormone measurements by radioimmunoassay (RIA) and enzyme immunoassay (EIA) [58]. Although steroids were measured using different techniques in different field seasons, the relative values demonstrated consistent trends. Stimulated ovarian T production was reduced through the urban area in the fall (Fig 5B). Exposure to MWWE was not found to impact stimulated ovarian steroid production of T in the spring (Fig 5D). E2 production was found to be reduced downstream of the Waterloo and Kitchener MWWTPs in 2 of 3 years in the fall (Fig 5A) and was lower downstream of the Kitchener plant in 1 of 2 years in the spring compared with E2 production in the reference site (Fig 5C). Stimulated testicular production of 11KT and T was found to be lower at sites downstream of the Kitchener and Waterloo MWWTPs in fall and spring in most years (Fig 6). While the size of the impact varied between treatment plants and years, there was a consistent trend of suppression of androgen production downstream of the Waterloo and Kitchener MWWTPs compared with the reference (R) site.

### Histology

**Fig 7:**
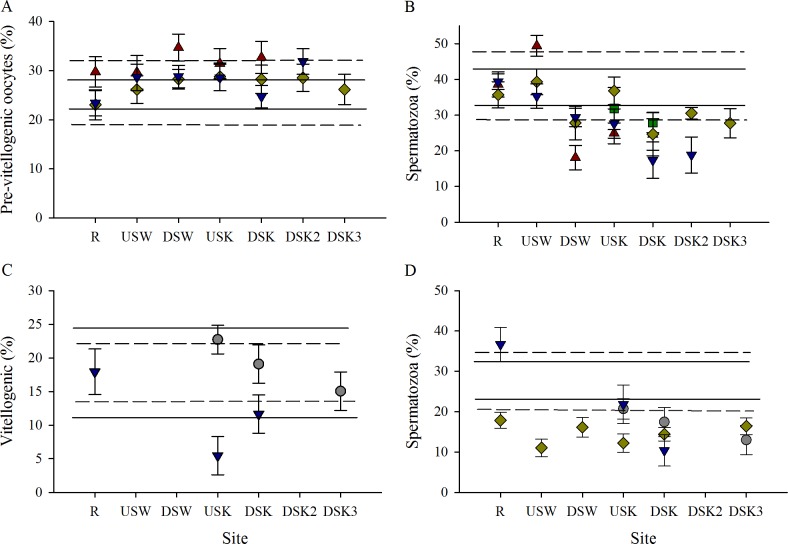
Impact of MWWE on gonad development is clear in males, but not females. Proportion of advanced cell types in (A, C) female and (B, D) male gonads of rainbow darter collected through an urban gradient. The proportion of all cell types was assessed through histological analysis of female and male gonads collected in (A, B) fall and (C, D) spring field seasons in 2007 (red triangles), 2009 (grey circles), 2010 (green squares), 2011 (yellow diamonds), and 2012 (blue down-facing triangles). The dashed line indicates the upper 25% critical effect size calculated from the mean of the data from the rural reference site (R), and the solid line indicates the upper 95% confidence interval from the pooled R.

While multiple categories of stages of gonad development were assessed in this study, data for only the most advanced stages are presented in this section. The relative proportion of other stages is presented in S1–S4 Figs. Ovarian development was found to be moderately impacted by urbanization (Fig 7A and 7C). An increase in the development of ovarian follicles was observed through the urban area in the fall with an increase in the proportion of pre-vitellogenic oocytes (Fig 7A). Additionally, there was a moderate decrease in the proportion of vitellogenic oocytes in the urban area in the spring (Fig 7C); however, no clear impact of MWWE was observed. During the fall field collections, sperm development was consistently delayed in fish collected from sites downstream of MWWTPs, with a lower proportion of spermatozoa (Fig 7B). The proportion of spermatozoa in spring 2009 was found to be unchanged through the gradient. In spring 2011, the proportion of spermatozoa was found to be lower at the site upstream of the MWWTPs but not downstream when compared with the R site (Fig 7D).

**Fig 8:**
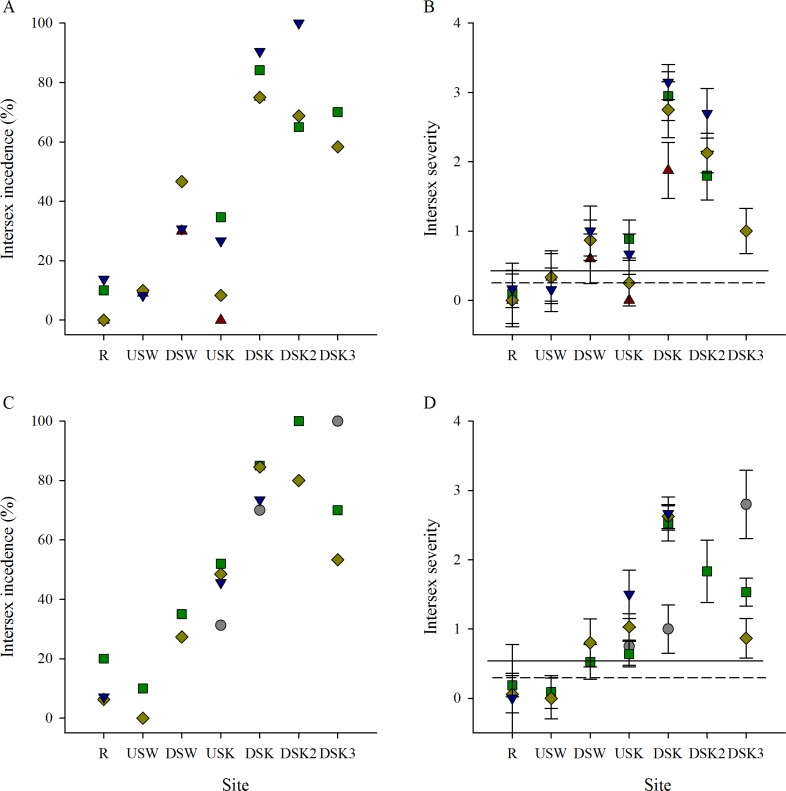
Intersex incidence and severity are consistently higher at sites downstream of MWWTP outfalls. Intersex (A, C) incidence and (B, D) severity of male rainbow darter collected through an urban gradient in the (A, B) fall of 2007 (red triangles), 2009 (grey circles), 2010 (green squares), 2011 (yellow diamonds), and 2012 (blue down-facing triangles) and in the (C, D) spring of 2009 (grey circles), 2010 (green squares), 2011 (yellow diamonds), and 2012 (blue down-facing triangles). The dashed line indicates the upper 25% critical effect size calculated from the mean of the data from the rural reference site (R), and the solid line indicates the upper 95% confidence interval from the pooled R.

Intersex incidence and severity were consistently higher at sites downstream of the Waterloo and Kitchener MWWTPs than at upstream sites and the R site in the fall field seasons (Fig 8A and 8B). The spring field collections demonstrated an increase in intersex incidence and severity through the urban region, with additional increases downstream of MWWTPs (Fig 8C and 8D).

### Somatic indices

**Fig 9:**
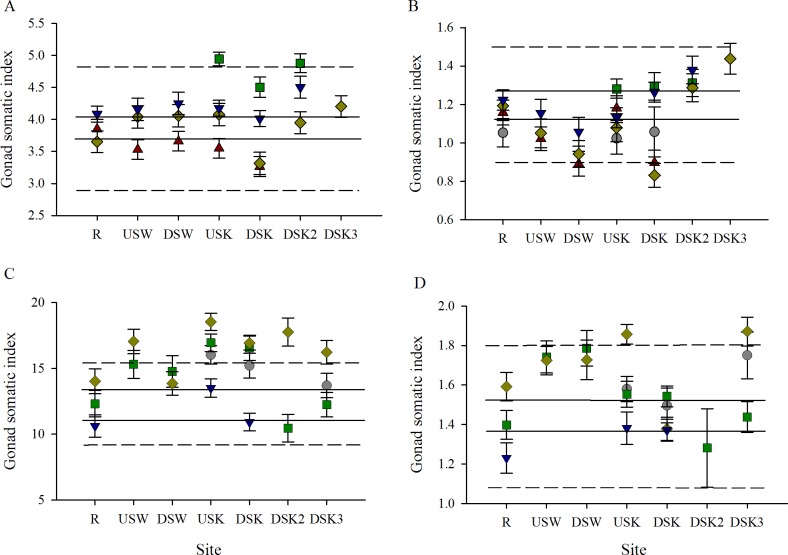
Changes in gonad somatic index through the urban gradient are variable and difficult to interpret. Gonad somatic index of (A, C) female and (B, D) male rainbow darter collected through an urban gradient in the (A, B) fall of 2007 (red triangles), 2009 (grey circles), 2010 (green squares), 2011 (yellow diamonds), and 2012 (blue down-facing triangles) and in the (C, D) spring of 2009 (grey circles), 2010 (green squares), 2011 (yellow diamonds), and 2012 (blue down-facing triangles). The dashed line indicates the 25% critical effect size calculated from the mean of the data from the rural reference site (R), and the solid line indicates the 95% confidence interval from the pooled R.

No direct impacts of MWWE on relative gonad size were observed in female rainbow darter. While some higher GSI measurements were detected in fall 2010, this occurred at sites throughout the urban area and seemed to be driven by annual differences, not site differences (Fig 9A). Female GSI was higher at sites upstream of MWWTPs in some years during spring collections; this effect persisted downstream in some subsequent years but dissipated downstream in other years (Fig 9C). In the fall, decreases in male GSI were found downstream of the Waterloo MWWTP (DSW) in all years, and inconsistent decreases were observed at the site downstream of the Kitchener MWWTP (DSK). These decreases approached biological significance (surpassing the 25% critical effect size) in fall 2007 and fall 2009 (Fig 9B). In contrast, GSI was statistically but not biologically higher at the second and third sites downstream of Kitchener MWWTP (DSK2 and DSK3) than at the R site in fall (Fig 9B). No consistent changes in male GSI were noted in the spring field collections (Fig 9D). Annual variability in GSI was greater at the R site during the spring than the fall for both males and females (Fig 9).

**Fig 10:**
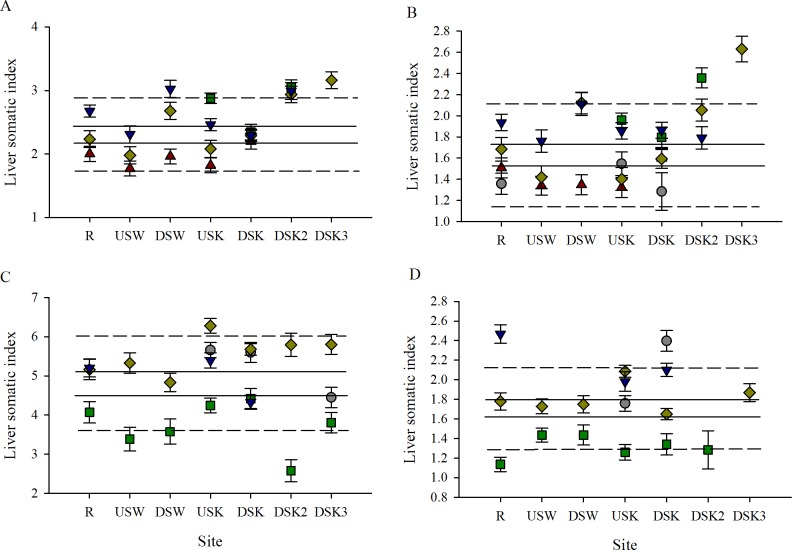
Liver somatic index is variable through the urban watershed. Liver somatic index of (A, C) female and (B, D) male rainbow darter collected through an urban gradient in the (A, B) fall of 2007 (red triangles), 2009 (grey circles), 2010 (green squares), 2011 (yellow diamonds), and 2012 (blue down-facing triangles) and in the (C, D) spring of 2009 (grey circles), 2010 (green squares), 2011 (yellow diamonds), and 2012 (blue down-facing triangles). The dashed line indicates the 25% critical effect size calculated from the mean of the data from the rural reference site (R), and the solid line indicates the 95% confidence interval from the pooled R.

In the fall field collections, the relative size of female livers increased modestly downstream of the Waterloo MWWTP (DSW) in fall 2011 and 2012 (but not 2007) and increased at the second and third sites downstream of the Kitchener MWWTP (DSK2 and DSK3) in the fall of all years (Fig 10A). No consistent trends in female LSI were observed in the spring (Fig 10C). Similar to the finding in females, male LSI increased at DSW in the fall of 2011 and 2012 (but not 2007). The increases in liver size downstream at the DSK2 and DSK3 sites were less consistent in males than females, with biologically significant changes observed in fall 2010 and 2011, but not 2012 (Fig 10B). No trends were observed in male LSI during the spring field collections (Fig 10D). Similar to GSI, the spring LSI at the R site was found to be highly annually variable in both sexes.

**Fig 11:**
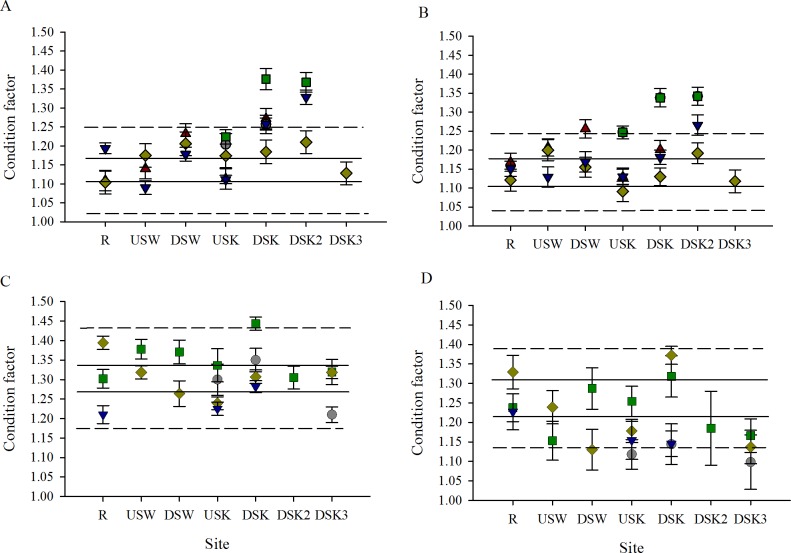
Condition factor increases downstream of MWWTP outfalls in fall, but not spring. Condition factor of (A, C) female and (B, D) male rainbow darter collected through an urban gradient in the (A, B) fall of 2007 (red triangles), 2009 (grey circles), 2010 (green squares), 2011 (yellow diamonds), and 2012 (blue down-facing triangles) and in the (C, D) spring of 2009 (grey circles), 2010 (green squares), 2011 (yellow diamonds), and 2012 (blue down-facing triangles). The dashed line indicates the 10% critical effect size calculated from the mean of the data from the rural reference site (R), and the solid line indicates the 95% confidence interval from the pooled R.

Condition factor was higher in females collected downstream of both MWWTPs in the fall in all years. This increase was only biologically significant in females collected at DSK and DSK2 in fall 2009, 2010, and 2012 (but not 2011; Fig 11A). In males, condition factor was also elevated at sites downstream of MWWTPs in the fall, but the response was less consistent. A statistically and biologically significant increase was found at DSW in fall 2007 (but not 2011 or 2012), at DSK in fall 2012 (but not 2009, 2011, or 2012), and at DSK2 in fall 2010 and 2012 (but not 2011) (Fig 11B). No clear changes in condition factor were observed in females or males between the reference (R) sites and sites downstream of MWWTPs during the spring field collection (Fig 11CD). The variability of condition at the R site was larger in the spring than the fall (Fig 11).

### Cluster analyses

**Fig 12:**
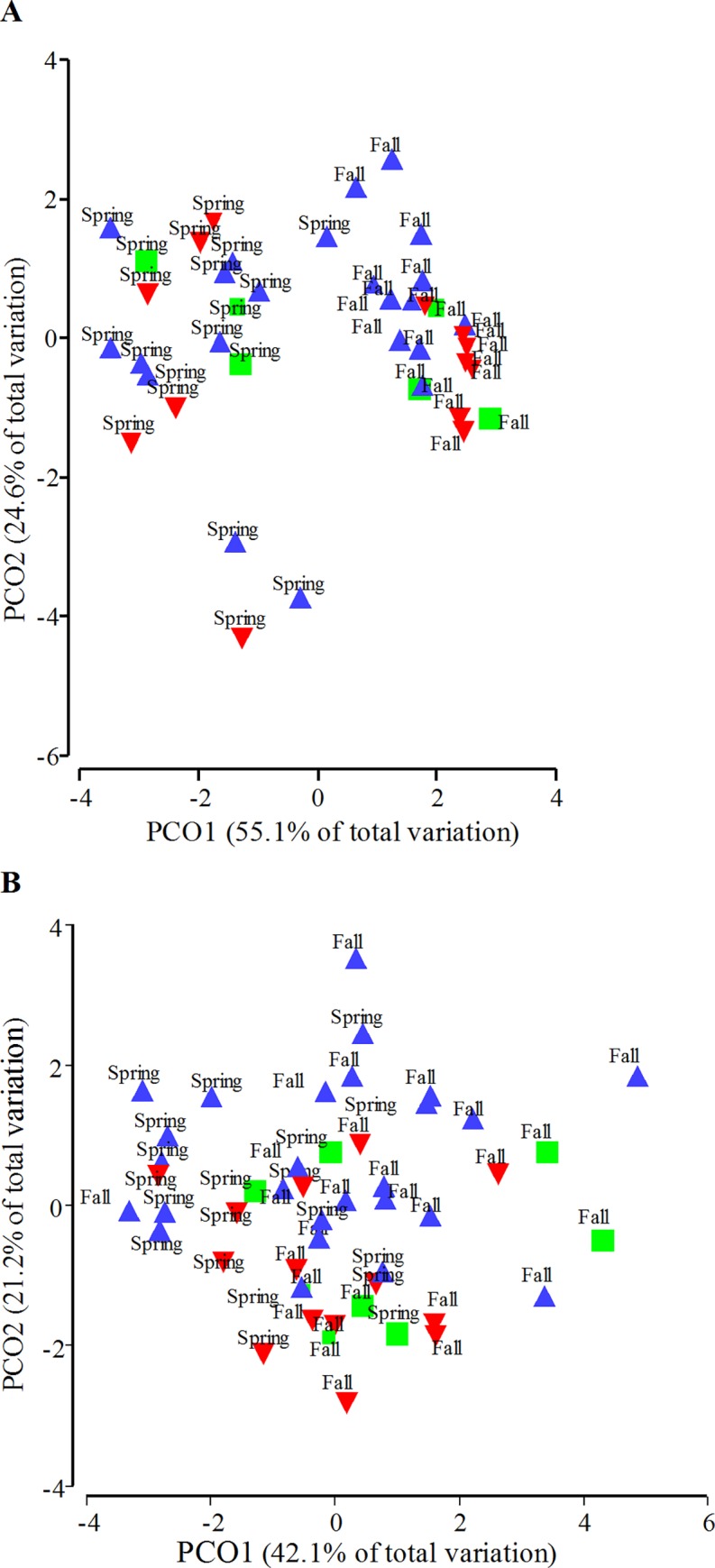
Principal coordinates (PCO) analysis of biological measures. (A) Female and (B) male biological measures collected from a reference site (green squares), sites upstream of municipal wastewater treatment plant (MWWTP) outfalls (red downward triangles), or downstream of MWWTP outfalls (blue triangles). Each sampling site is labeled with the season (spring or fall) that data were collected. PCO is based on a Euclidean distance resemblance matrix.

**Fig 13:**
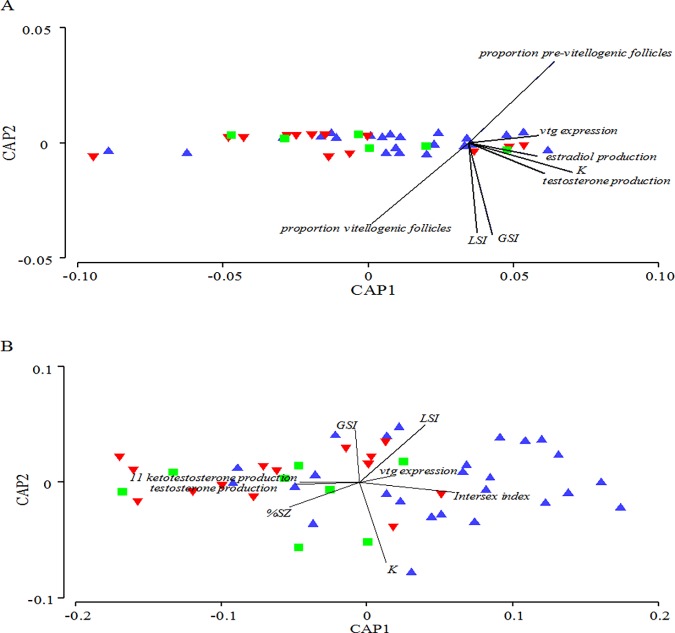
Canonical analysis of principal coordinates (CAP) ordination with biological variables as vectors. (A) Female and (B) male biological measures collected from a reference site (green squares), sites upstream of municipal wastewater treatment plant (MWWTP) outfalls (red downward triangles), or downstream of MWWTP outfalls (blue triangles).

Principal coordinates (PCO) analysis of female data demonstrated that there was distinct separation of biological variables between seasons. This separation occurred mainly by PCO1. Some separation of sites could be observed in fall data by PCO2, with downstream sites clustering more closely together and with some overlap between the reference site and upstream sites (Fig 12A). When a canonical analysis of principal coordinates (CAP) test was performed on female data, it was found that there were no significant variables that explained a majority of the separation (Fig 13A). PCO analysis of male biological endpoints exhibited less distinction between seasons than that of the female endpoints (which separated along PCO1) (Fig 12B). A separation of the sites along PCO2 was noted with exposed (downstream) sites grouping together (Fig 12B). The CAP analysis revealed that site separation in males was correlated with intersex severity (*r* = 0.786) along CAP1, and some slight separation along CAP2 was noted as well, which was correlated with somatic indices (*r* = 0.55, 0.59 and -0.84 for GSI, LSI, and K respectively) (Fig 13B).

Somatic indices were excluded from DISTLM analyses due to their variable nature. All other biological measures were included. Female biological measures were associated with carbamazepine, but no other pharmaceutical (Table C in S1 File). In contrast, male biological measures were associated with all pharmaceuticals except for carbamazepine (Table C in S1 File).

### Consistency / variability

In females, the interpretation of whether or not an impact of MWWE was observed on certain endpoints was dependent on the reference site chosen. Changes in measures at lower levels of biological organization were present when compared with the rural reference site (R) but not as clearly present when compared with the site directly upstream of the MWWTPs (USW, USK; Table 3). The consistency between years of the presence or absence of a response when compared with the R site was greater in the fall than in the spring, and more consistent downstream of the Waterloo MWWTP than the Kitchener MWWTP (Table 3). In males, there was some inconsistency in interpretation depending on which reference site was used for the comparison (rural reference (R) versus direct upstream reference (USW, USK)), but not to the same extent as in females (Table 4). The consistency of male responses to MWWE between years was greater in the fall than the spring (Table 3). While responses of males downstream of the Waterloo MWWTP were more consistent than the responses of males downstream of the Kitchener MWWTP in fall, this relationship reversed in the spring field collections.

**Table 3:** Comparison of significant changes observed in female rainbow darter downstream of the Waterloo (DSW) and Kitchener (DSK) MWWTPs in relation to either the rural reference site (R) or the immediate upstream (USW and USK, respectively) reference site. Comparisons are made for fall and spring field collections.

Female endpoints	# years in which differences were observed in fall				# years in which differences were observed in spring			
Downstream site	DSW		DSK		DSW		DSK	
Reference site	R	USW	R	USK	R	USW	R	USK
Gene expression	1/2	0/2	0/2	0/2	NA	N/A	0/1	N/A
Testosterone production	2/2	0/2	3/3	0/2	1/1	0/1	2/3	2/2
Estradiol production	3/3	1/3	2/2	1/3	0/1	1/1	1/2	0/2
Ovarian development	1/3	0/3	1/3	0/3	NA	NA	0/2	0/2
GSI	0/3	0/3	0/4	2/4	0/2	1/2	2/4	1/4
LSI	2/3	2/3	0/4	2/4	0/2	0/2	0/4	2/4
K	0/3	2/3	4/5	3/5	0/2	0/2	1/4	3/4

**Table 4:** Comparison of significant changes observed in male rainbow darter downstream of the Waterloo (DSW) and Kitchener (DSK) MWWTPs in relation to either the rural reference site (R) or the immediate upstream (USW, USK respectively) reference site. Comparisons are made for fall and spring field collections.

Male endpoints	# years in which differences were observed in fall				# years in which differences were observed in spring			
Downstream site	DSW		DSK		DSW		DSK	
Reference site	R	USW	R	USK	R	USW	R	USK
Gene expression	1/2	2/2	1/3	1/2	N/A	N/A	1/1	N/A
Testosterone production	2/3	2/3	3/3	2/3	1/1	1/1	2/3	1/2
11-ketotestosterone production	2/3	2/3	3/3	1/3	1/1	1/1	1/2	0/2
Testis development	3/3	2/3	4/4	2/4	0/1	0/1	3/3	1/3
Intersex severity	3/3	1/3	4/4	4/4	1/2	1/2	4/4	3/4
GSI	1/3	0/3	2/5	3/5	0/2	0/2	0/4	1/4
LSI	2/3	2/3	0/5	1/5	0/2	0/2	1/4	2/4
K	1/3	0/3	1/4	2/4	1/2	2/2	0/4	1/4

**Fig 14:**
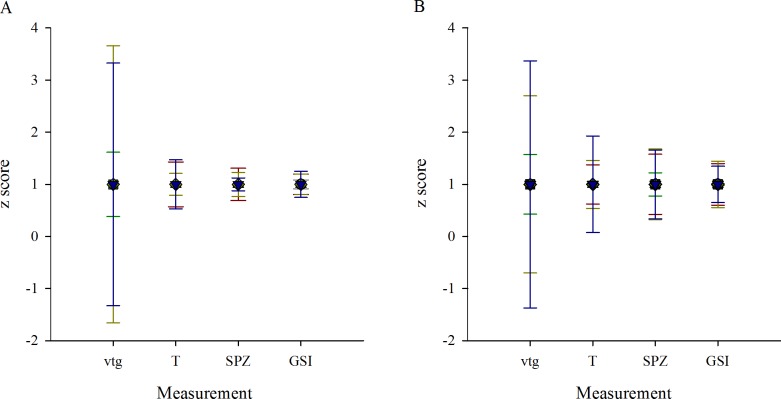
Variability of biological measures decreases with increasing biological complexity. A comparison of variability in measures of male rainbow darter reproduction across levels of biological organization at a (A) reference site and a (B) MWWE-exposed site from collections in the fall of 2007 (red triangles), 2010 (green squares), 2011 (yellow diamonds), and 2012 (blue down-facing triangles). Measures compared include gene expression of *vtg* (VTG), *in vitro* production of testosterone (T), gonad development and relative proportion of spermatozoa (SPZ), and gonad somatic index (GSI).

To determine the amount of variability within a site/season within each endpoint, data were normalized so that the mean equaled 1 within the site. The standard deviation then represented the amount of variability in each measure within a year/season/site. Variability of endpoints was found to decrease in measures as they increased in complexity in terms of biological organization (Fig 14A). This was true at the R site as well as at the DSK site (Fig 14). While the variability of gene expression was comparable between sites, the variability of other measures was greater at the DSK site than at the R site (Fig 14).

## Discussion

This study is among the few to examine the variability in the response of wild fish to watershed stressors, including municipal wastewater effluents, across levels of biological organization in multiple seasons and years. While field collections can often be difficult to interpret due to the many confounding factors and the presence of multiple stressors, we found that the use of endpoints across multiple levels of biological organization allowed us to more clearly identify stressors and assess their impact. Through the analysis of multiple endpoints across levels of biological organization and over multiple seasons and years, we concluded that MWWE has impacts on reproduction in male rainbow darter. The degree of the reproductive disruption and the number of biological measures affected did vary between sexes, seasons, and years. The largest differences in the responses were found to be associated with season. Additionally, the consistency of the reproductive disruption and variability differed for each of the selected measures across the levels of biological organization. Measures at the tissue level, such as gonad development and presence of intersex, were the most consistent and were also the most useful endpoints in discerning the effects of point sources such as wastewater outfalls. In the following sections the general patterns in the measures are first described and then the seasonal variability and consistency/variability of biological measures are explored.

### Impacts associated with the urban gradient and wastewater outfalls (spatial changes)

A clear change in surface water chemistry in response to urbanization, especially associated with wastewater outfalls, was observed in this study. The central Grand River receives high levels of nitrogenous waste. While some of the nitrate in the river originates from upstream (probably associated with intensive agriculture), there was a clear increase in ammonia and nitrate downstream of the 2 MWWTP outfalls. The pharmaceuticals analyzed in this study followed a trend similar to that of the nitrogenous compounds (i.e., ammonia, nitrate), with large increases directly downstream of the MWWTP outfalls. This is to be expected, as MWWE is well known to be a source of CECs. While some of the chemicals we measured disappeared quickly downstream (e.g., triclosan), others appear to persist for many kilometres downstream (i.e., carbamazepine, venlafaxine). Through the use of models, Arlos *et al*. [59] demonstrated that while some chemicals (like triclosan) are probably removed through photolysis, the concentrations of other chemicals are decreased primarily through dilution or attenuation. Since the volume of effluent is relatively consistent, the river flow plays an important role in modifying exposure temporally. The data presented in this study consist of grab samples and are highly dependent on the weather and resulting flows immediately before sampling and thus may not accurately reflect the exposure of fish during critical life stages.

The impact of MWWE on female reproductive health was not clear in this study. Overall, some subtle differences between sites were observed in the fall field collections but not in the spring (as demonstrated in Fig 12A). When we examined the individual biological markers across levels of biological organization, we observed few consistent and clear changes associated with MWWE. For instance, although synthesis of ovarian estradiol and testosterone was usually lower near the MWWTPs in spring and fall than at the rural reference site (R), few differences were noted between directly upstream and downstream sites in either season. Similarly, increases in the proportion of pre-vitellogenic cells were observed in the fall downstream of the 2 MWWTPs when compared with the R site but not the USW or USK sites. Additionally, the proportion of vitellogenic oocytes was lower downstream of the Kitchener MWWTP than at the R site during 1 spring season. While these observations were inconsistent, the direction of the changes is suggestive of long-term exposure to estrogenic compounds. The lower proportion of vitellogenic oocytes in the spring, however, could also be explained by differences in the timing of spawning. Although we did not observe ovulated eggs in the ovarian cavity, it is possible that fish spawned earlier at sites near the MWWE. Other field studies of MWWE impacts have reported changes in ovarian development and noted delayed ovarian maturation [60], increased oocyte atresia [61], interruption of spawning [62], as well as decreased plasma estradiol and testosterone concentrations [43]. In the laboratory, exposure to MWWE has been shown to induce atresia of ovarian follicles [63], alteration of ovarian development [63], and reduction of egg laying in fishes [64–67]. Although these studies suggest that there are potential negative impacts of MWWE on females, we found these hard to separate from the effects of other stressors in the wild. The changes observed in females in this study tended to occur both upstream and downstream of the MWWTPs, suggesting that any impacts present may have been due to cumulative urban effects rather than just MWWE.

Male reproductive health measures responded to MWWE exposure in both seasons and at most levels of biological organization. The PCOS clearly demonstrated a separation of sites based on the biological endpoints (Fig 12B), which were indicative of MWWE exposure. Male *vtg* expression increased directly downstream of the treatment plants in some years. Increases in *vtg* expression have been associated with the presence of estrogenic compounds [68–70] and are frequently found downstream of MWWTPs [71–73]. Male gonad sex steroid production and gonad development were found to be decreased and delayed, respectively. Additionally, a consistent increase in the occurrence and severity of intersex was found downstream of MWWE outfalls in the fall. These measures clearly demonstrate exposure to MWWE, resulting in endocrine disruption and impaired sexual development. Similar to the increased expression of *vtg*, these endpoints are all suggestive of the presence of estrogenic compounds. The estrogenicity of the 2 MWWTPs in this study was assessed using the yeast estrogen screen (YES) in a previous study. The concentrations of estradiol equivalents in treated effluent in that study were found to be 4.3 ± 0.07 and 16.99 ± 0.40 ng/L in the Waterloo and Kitchener wastewater, respectively [48].

### Consistency of interpretation between seasons

While there were fewer data available for spring field collection than fall, we generally found greater consistency in the interpretation of a response in the fall than in the spring. This is partly due to the high annual variability in most measures in the spring field season compared with the fall. The somatic indices demonstrate this phenomenon most clearly, with large variation between years at the R site. There are several explanations for the increased variation in somatic indices during the spring. First, the variability between years in spring LSI could be due to differences in habitat (e.g., temperature, flow, food availability). Rainbow darter rapidly increase the size of their gonad and liver during the period just before spawning, a process that is dependent on food availability [74] and thus could be altered by variations in weather patterns. Second, the timing of sampling (during this biologically dynamic period) in the spring can be variable because access to sample sites is dependent on river flow, which is elevated by snow melt. Third, the interpretation of the impacts of MWWE may differ between seasons because of the increased fish movement in the spring, as rainbow darter move larger distances in the early spring than in the fall or summer. The increase in movement during the spring is probably related to locating ideal spawning sites.[75]. Increased movement during spring and summer would explain the higher levels of intersex incidence and severity above the Kitchener MWWTP in spring than in fall.

In terms of developing a biological monitoring program for CECs, this study demonstrates how an understanding of the indicator species is essential to developing an effective program. In a northern climate, small-bodied species such as darters that spawn in early spring are difficult to consistently sample, due to the aforementioned influences of variable weather conditions and movement associated with spawning. It is therefore strongly recommended that sampling occur in late fall when gonad development and physiology are more consistent. Barrett and Munkittrick [76] reached a similar conclusion based on an assessment of the extensive Environmental Effects Monitoring program results in Canada.

### Consistency/variability of measurements (across levels of biological organization)

When examining the impacts of MWWE, we found that measures at the lowest end of the biological scale (i.e., gene expression, Fig 4) were less consistent in their responses between years and were the most variable endpoint in terms of the range of data collected within a field season/site. Measures in the middle of the biological scale (i.e., intersex, Fig 8) were more consistent between seasons and years and also varied less within a year. While measures at the highest biological level measured (i.e., somatic indices; Figs 9–11) were the least consistent among years, they also varied the least within a season (Fig 12, Tables 3 and 4). We observed variation in the data sets not only across levels of biological organization but also across space. Measures in the middle and at the upper end of the biological scale were more variable at the exposed site than the R site (Fig 12).

The high variability in gene expression within a site/year was not unexpected. Gene expression is a dynamic process that changes hourly in response to the ever-changing physiological demands of an organism. While an increase in variability within a site can provide us with information about the nature of the response (exposure, movement, etc.), it can be problematic for interpretation within a biological monitoring program. A clear increase in the expression of *vtg* in the liver of male rainbow darter downstream of the MWWTPs was present in some years; however, in other years this response was blunted or absent. Although this could be due to fluctuations in the composition of MWWE, or the amount of dilution of MWWE, not enough is known about the variability in the *vtg* response of a chronically exposed fish to draw any conclusions about this observation.

Somatic indices were the least variable endpoint we measured in terms of the standard deviation at a site. They were also the least consistent endpoint between years of collections. It is difficult to know if the annual changes in the site-specific patterns of somatic indices are associated with abiotic fluctuations (exposure, flow, effluent quality, oxygen, etc.) or natural variability. In some years, fish may be exposed during a critical life stage to specific contaminants, or there may be extreme conditions (i.e., drought) that result in changes in somatic indices. Because somatic indices are more likely to be associated with general stressors or food availability, they may respond differently than endpoints that are more mechanistically linked to exposure to CECs in MWWE (e.g., intersex). High annual variability in select biomarkers has been observed in other studies as well [77]. While it has been suggested that the use of multiple reference sites may alleviate this variability [78], another study has found that this variability is reduced only when more than 4 reference sites, or 4 years of data collected from 1 site, are assessed [79]. The results from our study demonstrate how dynamic somatic indices can be between years in a small-bodied fish like darters and show the risk of over-interpreting them when only 1 year of sampling is completed or taken out of context of other endpoints. Many studies in the literature rely on this type of minimal sampling design and do not fully consider annual variability. For example, our own studies previously reported differences in somatic indices at these same sites and attributed them to wastewater exposure [48].

In contrast to the high variability of gene expression and the low consistency of somatic indices, the variability of histological measures was low and the consistency of these measures was high. Gonad development in males was delayed in most fall field collections (but not spring). Additionally, the intersex incidence and severity were the most consistent measure in this study across years (in all the fall field seasons and most spring field seasons). Similarly, a study of male smallmouth bass (*Micropterus dolomieu*) found that the incidence of intersex was similar in 2 years of collection [43]. While smallmouth bass intersex incidence and severity were comparable between spring and fall, the measures were both lower at sites in the summer [36, 43].

This examination of variability and consistency in endpoints across levels of biological organization provides contrasting messages about the appropriate design of a biological monitoring program. While it seems that it is necessary to collect multiple years of data to assess the impacts of MWWE on somatic indices, individual measures, such as intersex, are fairly consistent among years and would not require multiple years of biological monitoring. Similarly, while measures at the low end of the biological scale (gene expression) are highly variable, measures at higher levels of biological organization (somatic indices) are less variable. This must be considered in the design and when applying statistics to the data sets generated. For endpoints with large variability the sample size must be increased to be able to detect change. Much larger sample sizes will be needed for measures of gene expression than for measures of somatic indices. However, this is seldom considered in monitoring designs or studies and often, because of the cost or other consideration (e.g., sample availability), sample size is not optimized for each endpoint. Additionally, a larger sample size may not be practical for a few reasons: because of the amount of fishing effort required, because removing additional fish from an affected area could have negative impacts on the population, and because it is difficult to produce an ethical justification for using larger numbers of fish to detect minimal change.

### The population-level conundrum

The largest gap in this study is the inability to link the observation that male rainbow darter have impaired reproductive health to a higher level endpoint, namely implications for population sustainability. While measures of sex ratio were taken in some years, it was determined that the habitat preferences of males and females differed and changed across seasons, thus making these data inconclusive. The lack of population-level endpoints is a major weakness in most studies that evaluate the impacts of CECs from MWWE. This is a significant issue because measures at higher levels of biological organization provide regulators with the information necessary to make decisions. For example, a recently proposed framework for screening sites at risk from CECs suggested identifying sites where population and community effects are observed [80].

Despite the importance of these measures, the assessment of population-level endpoints in response to CECs proves to be problematic for a couple of reasons. Firstly, there are few standardized methods that test for population-level responses to CECs. This was recently addressed in a review by Hamilton *et al*. [81]. The authors suggested several manners in which population-level effects could be assessed with modern technology. A second reason why assessing population-level endpoints are problematic is the amount of variability. We were unable to associate changes in somatic indices with a specific stressor in our multi-stressor system, despite there being strong indications of an effect related to endocrine disruption. It is likely that it will be even more difficult to detect change in measures at the population, community or ecosystem levels and directly associate them with CECs.

## Conclusion

This study supports the use of the hierarchical biological system in biological monitoring programs. Making linkages to specific stressors (e.g., effluents) is strengthened by this approach, but natural variability and complexity of stressors (e.g., effluents) and the environment (e.g., flow, habitat) make it difficult to establish cause and effect (e.g., predictive) relationships. This study also cautions against the over-interpretation of monitoring data that do not consider the lifecycle of the sentinel species and the implications of natural variability (spatial and temporal). By placing our results in the context of an adverse outcome pathway we can better understand the associations between individual- and population-level impacts of MWWE and the thresholds of these impacts.

## Supporting Information

S1 File Table A-C.
(PDF)

S1 Fig Changes in ovarian development through an urban gradient in fall collections. Relative proportion of primary (A), cortical alveolar (B) and pre-vitellogenic oocytes represented by the mean (± SE). Assessment was determined through histological analysis of female gonads collected in fall field season of 2007 (red triangles), 2011 (yellow diamonds), and 2012 (blue down-facing triangles). The dashed line indicates the upper 25% critical effect size calculated from the mean of the data from the rural reference site (R), and the solid line indicates the upper 95% confidence interval from the pooled R.
(TIF)

S2 Fig Changes in ovarian development through an urban gradient in spring collections. Relative proportion of primary (A), cortical alveolar (B), pre-vitellogenic (C), and vitellogenic (D) oocytes represented by the mean (± SE). Assessment was determined through histological analysis of female gonads collected in spring field season of 2009 (grey circles) and 2012 (blue down-facing triangles). The dashed line indicates the upper 25% critical effect size calculated from the mean of the data from the rural reference site (R), and the solid line indicates the upper 95% confidence interval from the pooled R.
(TIF)

S3 Fig Changes in testis development through an urban gradient in fall collections. Relative proportion of spermatogonia (A), spermatocytes (B), spermatids (C), and spermatozoa (D) represented by the mean (± SE). Assessment was determined through histological analysis of male gonads collected in fall field season of 2007 (red triangles), 2010 (green squares), 2011 (yellow diamonds), and 2012 (blue down-facing triangles). The dashed line indicates the upper 25% critical effect size calculated from the mean of the data from the rural reference site (R), and the solid line indicates the upper 95% confidence interval from the pooled R.
(TIF)

S4 Fig Changes in testis development through an urban gradient in spring collections. Relative proportion of spermatogonia (A), spermatocytes (B), spermatids (C), and spermatozoa (D) represented by the mean (± SE). Assessment was determined through histological analysis of male gonads collected in spring field season of 2009 (grey circles), 2011 (yellow diamonds), and 2012 (blue down-facing triangles). The dashed line indicates the upper 25% critical effect size calculated from the mean of the data from the rural reference site (R), and the solid line indicates the upper 95% confidence interval from the pooled R.
(TIF)
