# Supplementary material for: An Assessment of the Spatial and Temporal Variability of Biological Responses to Municipal Wastewater Effluent in Rainbow Darter (Etheostoma caeruleum) Collected along an Urban Gradient
Source: PLoS One. 2016 Oct 24;11(10):e0164879. doi: 10.1371/journal.pone.0164879 (PMC5077097; doi:10.1371/journal.pone.0164879)
Supplement: S1 File — Table A-C. (PDF) [file pone.0164879.s001.pdf]

S1 File

Table A. Sample sizes of biological endpoints including analysis of gene expression of vitellogenin (*vtg*), stimulated in vitro steroid production of testosterone (T), 11-ketotestosterone (11KT), or 17β-estradiol (E2), histological analysis of gonad development (GD), incidence and severity of intersex (IS), relative proportion of gonad (GSI), relative proportion of liver (LSI), and condition factor (K).

Year / Season	*Site*	*Male*							*Female*					
		vtg	*T*	*11KT*	*GD*	*IS*	*GSI LSI*	*K*	*vtg*	*E2*	*T*	*GD*	*GSI LSI*	*K*
2007/ Fall	R	NA	8	8	5	10	20	20	NA	5	5	10	20	20
	USW	NA	10	11	5	11	22	23	NA	7	7	8	20	20
	DSW	NA	9	9	5	10	20	20	NA	9	9	10	20	20
	USK	NA	7	7	5	10	20	20	NA	6	7	10	20	20
	DSK	NA	9	9	5	8	20	20	NA	5	5	9	19	19
2009/ Fall	R	NA	NA	NA	NA	NA	19	NA	NA	NA	NA	NA	NA	NA
	USK	NA	NA	NA	NA	NA	15	NA	NA	NA	NA	NA	NA	NA
	DSK	NA	NA	NA	NA	NA	6	NA	NA	NA	NA	NA	NA	NA
	DSK2	NA	NA	NA	NA	NA	NA	NA	NA	NA	NA	NA	NA	NA
	DSK3	NA	NA	NA	NA	NA	NA	NA	NA	NA	NA	NA	NA	NA
2009/ Spring	USK	NA	17	17	10	16	37	37	NA	11	18	18	35	35
	DSK	NA	9	9	5	10	20	20	NA	6	9	10	20	20
	DSK3	NA	6	6	5	5	11	11	NA	7	10	10	20	20
2010/ Spring	R	NA	NA	NA	NA	42	42	42	NA	NA	NA	NA	18	18
	USW	NA	NA	NA	NA	22	22	22	NA	NA	NA	NA	15	71
	DSW	NA	NA	NA	NA	19	19	19	NA	NA	NA	NA	13	31
	USK	NA	NA	NA	NA	36	36	36	NA	NA	NA	NA	39	80
	DSK	NA	NA	NA	NA	19	19	19	NA	NA	NA	NA	20	169
	DSK2	NA	NA	NA	NA	6	6	6	NA	NA	NA	NA	17	15
	DSK3	NA	NA	NA	NA	30	30	30	NA	NA	NA	NA	20	62
2010/ Fall	R	10	NA	NA	NA	10	NA	NA	NA	NA	NA	NA	NA	NA
	USK	NA	NA	NA	5	26	40	40	NA	NA	NA	NA	40	40
	DSK	10	NA	NA	5	20	20	30	NA	NA	NA	NA	18	18
	DSK2	NA	NA	NA	5	20	21	21	NA	NA	NA	NA	21	21
	DSK3	10	NA	NA	NA	11	NA	NA	NA	NA	NA	NA	NA	NA
2011/ Spring	R	8	11	11	17	17	30	30	8	10	10	NA	20	63
	USW	NA	12	12	14	14	30	30	NA	10	10	NA	20	71
	DSW	NA	10	9	11	10	15	20	NA	11	11	NA	25	31
	USK	NA	19	20	13	33	62	62	NA	20	17	NA	40	80
	DSK	8	20	21	23	40	66	105	8	30	30	NA	63	169
	DSK2	NA	NA	NA	NA	NA	NA	NA	NA	10	10	NA	15	15
	DSK3	NA	14	14	15	15	29	29	NA	10	10	NA	21	62
2011/ Fall	R	9	10	10	5	9	14	14	7	8	NA	10	16	16
	USW	9	10	10	5	9	15	15	8	10	NA	12	15	15
	DSW	12	10	12	5	15	17	15	8	10	NA	13	15	15

	USK	9	10	10	5	12	16	17	8	8	NA	12	15	15
	DSK	12	10	10	5	8	22	22	9	10	NA	9	15	15
	DSK2	8	10	10	5	16	16	16	9	10	NA	12	16	16
	DSK3	NA	10	10	5	12	13	13	NA	10	NA	10	16	16
2012/ Spring	R	NA	10	9	5	16	26	26	NA	9	8	7	25	67
	USK	NA	6	9	5	15	23	23	NA	10	10	10	36	120
	DSK	NA	4	7	5	23	49	49	NA	10	10	10	41	167
2012/ Fall	R	9	20	21	5	18	30	30	8	19	16	14	30	69
	USW	8	8	8	5	13	16	16	7	14	14	13	15	46
	DSW	12	12	12	5	10	15	15	7	12	14	14	15	44
	USK	8	15	15	5	15	30	30	12	21	15	13	30	120
	DSK	13	20	20	5	20	31	31	7	21	13	18	29	55
	DSK2	7	10	10	5	10	16	16	8	15	15	14	16	40

Table B. Concentration of steroid hormones (ng/g tissue) produced by gonad tissue stimulated with forskolin (F), or hCG (H) and measured by RIA (R) or EIA (E), as described in the methods section for *in vitro* steroid production.

Year / Season	*Site*	*Male*		*Female*	
		T	11KT	E2	T
2007 / Fall (F, R)	R	3.84 ± 0.58	ND	2.80 ± 0.74	0.79 ± 0.33
	USW	2.66 ± 0.38	ND	2.97 ± 0.45	0.88 ± 0.24
	DSW	1.96 ± 0.17	ND	2.59 ± 0.41	0.89± 0.22
	USK	2.64± 0.28	ND	2.39 ± 0.63	1.06 ± 0.30
	DSK	1.0 ± 0.21	ND	1.72 ± 0.31	0.90 ± 0.19
2009 / Spring (F, R)	USK	0.75 ± 0.09	0.82 ± 0.25	0.81 ± 0.13	0.46 ± 0.14
	DSK	0.77 ± 0.10	0.76 ± 0.12	0.98 ± 0.21	0.71 ± 0.16
	DSK3	0.51 ± 0.04	0.70 ± 0.12	0.82 ± 0.24	0.38 ± 0.01
2011 / Spring (F, R)	R	35.5 ± 3.9	49.1 ± 7.5	43.6 ± 5.4	29.7 ± 5.9
	USW	42.2 ± 3.8	87.7 ± 7.2	25.4 ± 5.4	8.3 ± 5.9
	DSW	18.6 ± 4.1	14.8 ± 8.3	41.3 ± 5.2	18.9 ± 5.6
	USK	30.8 ± 3.0	24.2 ± 5.5	29.8 ± 3.9	14.6 ± 4.5
	DSK	25.6 ± 2.9	26.8 ± 5.4	30.7 ± 3.1	26.1 ± 3.4
	DSK2	NA	NA	30.1 ± 5.4	10.7 ± 5.9
	DSK3	24.1 ± 3.5	24.3 ± 6.6	32.8 ± 5.4	20.0 ± 5.9
2011/ Fall (H, R)	R	49.6 ± 3.3	50.2 ± 6.2	8.3 ± 4.2	NA
	USW	59.8 ± 3.3	46.7 ± 5.6	8.2 ± 3.8	NA
	DSW	55.3 ± 3.3	63.7 ± 6.2	20.3 ± 3.8	NA
	USK	41.0 ± 3.3	45.7 ± 6.2	15.4 ± 4.2	NA
	DSK	27.8 ± 3.3	10.9 ± 6.2	7.1 ± 3.8	NA
	DSK2	32.4 ± 3.3	11.5 ± 6.2	11.0 ± 3.8	NA
	DSK3	46.9 ± 3.3	40.8 ± 6.2	18.7 ± 3.8	NA
2012 / Spring (H, E)	R	21.4 ± 4.1	43.2 ± 8.3	48.6 ± 5.7	25.7 ± 6.6
	USK	22.7 ± 5.3	34.2 ± 5.5	38.8 ± 5.4	75.0 ± 5.9
	DSK	6.9 ± 6.5	26.8 ± 5.4	39.5 ± 5.4	24.6 ± 5.9
2012/ Fall (H, E)	R	20.8 ± 2.3	17.1 ± 4.3	23.1 ± 2.7	1.22 ± 0.15
	USW	23.6 ± 3.7	27.7 ± 6.9	33.6 ± 3.2	0.81 ± 0.16
	DSW	10.8 ± 3.0	4.3 ± 5.6	13.7 ± 3.4	0.72 ± 0.16
	USK	11.9 ± 2.7	6.5 ± 5.0	12.9 ± 2.6	0.67 ± 0.15
	DSK	8.1 ± 2.3	2.4 ± 4.4	14.1 ± 2.6	0.62 ± 0.16
	DSK2	6.5 ± 3.3	2.9 ± 6.2	16.0 ± 3.1	0.88 ± 0.15

Table C. DISTLM analysis reveals that there is no association between select pharmaceuticals and female biological endpoints, but there is an association between some pharmaceuticals and male biological endpoints. Significance is indicated by * when $p<0.05$.

Sex	Pharmaceutical	SS	Pseudo-F	P	Proportion
Female	Naproxen	5.30	1.36	0.321	0.061
	Ibuprofen	1.39	0.341	0.688	0.016
	Venlafaxine	-0.953	-0.227	0.879	0.011
	Carbamazepine	16.8	5.01	0.020 *	0.193
	Triclosan	-2.78	-0.650	0.960	-0.032
Male	Naproxen	13.9	4.10	0.040 *	0.124
	Ibuprofen	19.9	6.29	0.009 *	0.178
	Venlafaxine	22.7	7.40	0.007 *	0.203
	Carbamazepine	10.6	3.05	0.086	0.095
	Triclosan	20.7	6.60	0.007 *	0.185
