# Supplementary figures and images for: An Assessment of the Spatial and Temporal Variability of Biological Responses to Municipal Wastewater Effluent in Rainbow Darter (Etheostoma caeruleum) Collected along an Urban Gradient
Source: PLoS One. 2016 Oct 24;11(10):e0164879. doi: 10.1371/journal.pone.0164879 (PMC5077097; doi:10.1371/journal.pone.0164879)

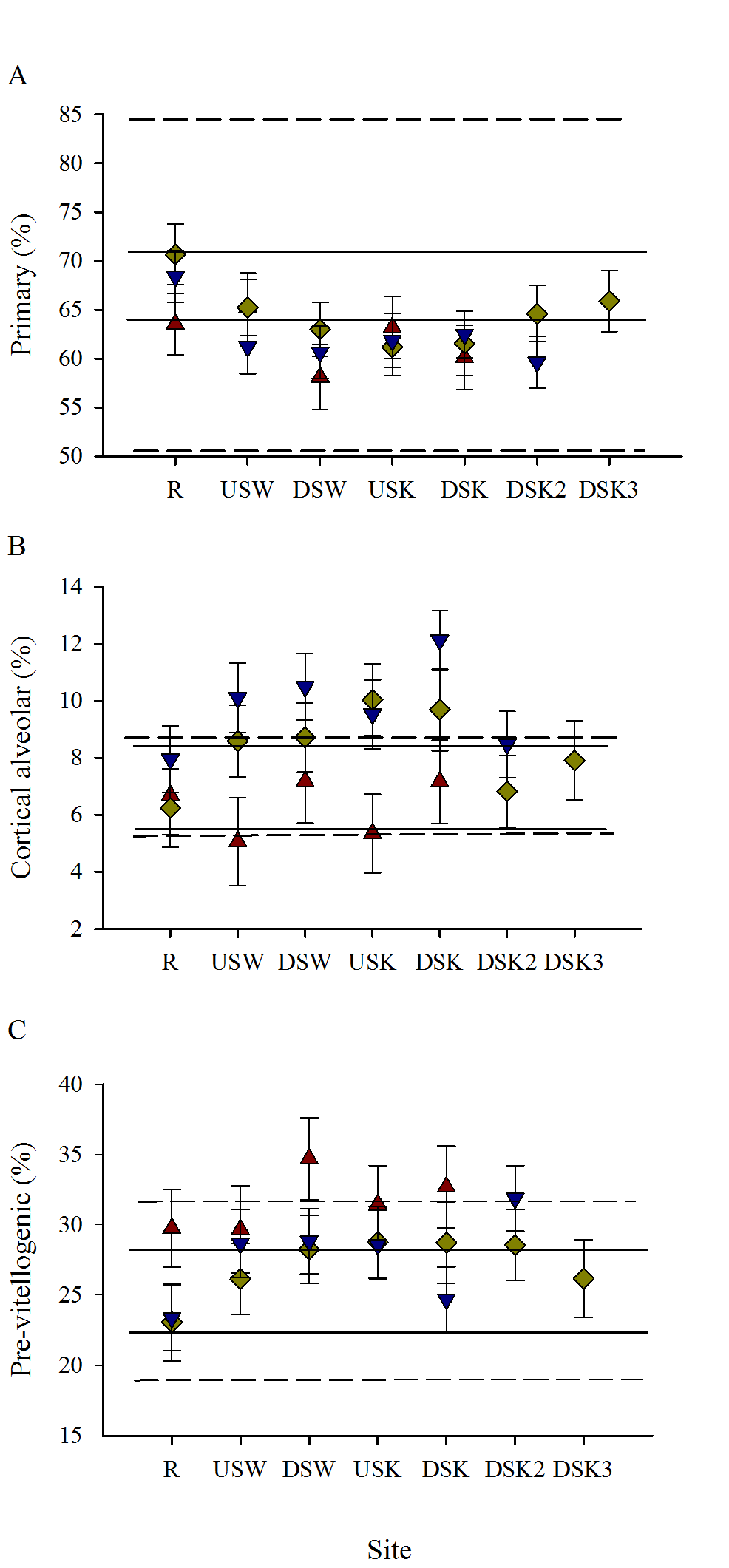

Supplement: S1 Fig — Relative proportion of primary (A), cortical alveolar (B) and pre-vitellogenic oocytes represented by the mean (± SE). Assessment was determined through histological analysis of female gonads collected in fall field season of 2007 (red triangles), 2011 (yellow diamonds), and 2012 (blue down-facing triangles). The dashed line indicates the upper 25% critical effect size calculated from the mean of the data from the rural reference site (R), and the solid line indicates the upper 95% confidence interval from the pooled R. (TIF) [file pone.0164879.s002.TIF]

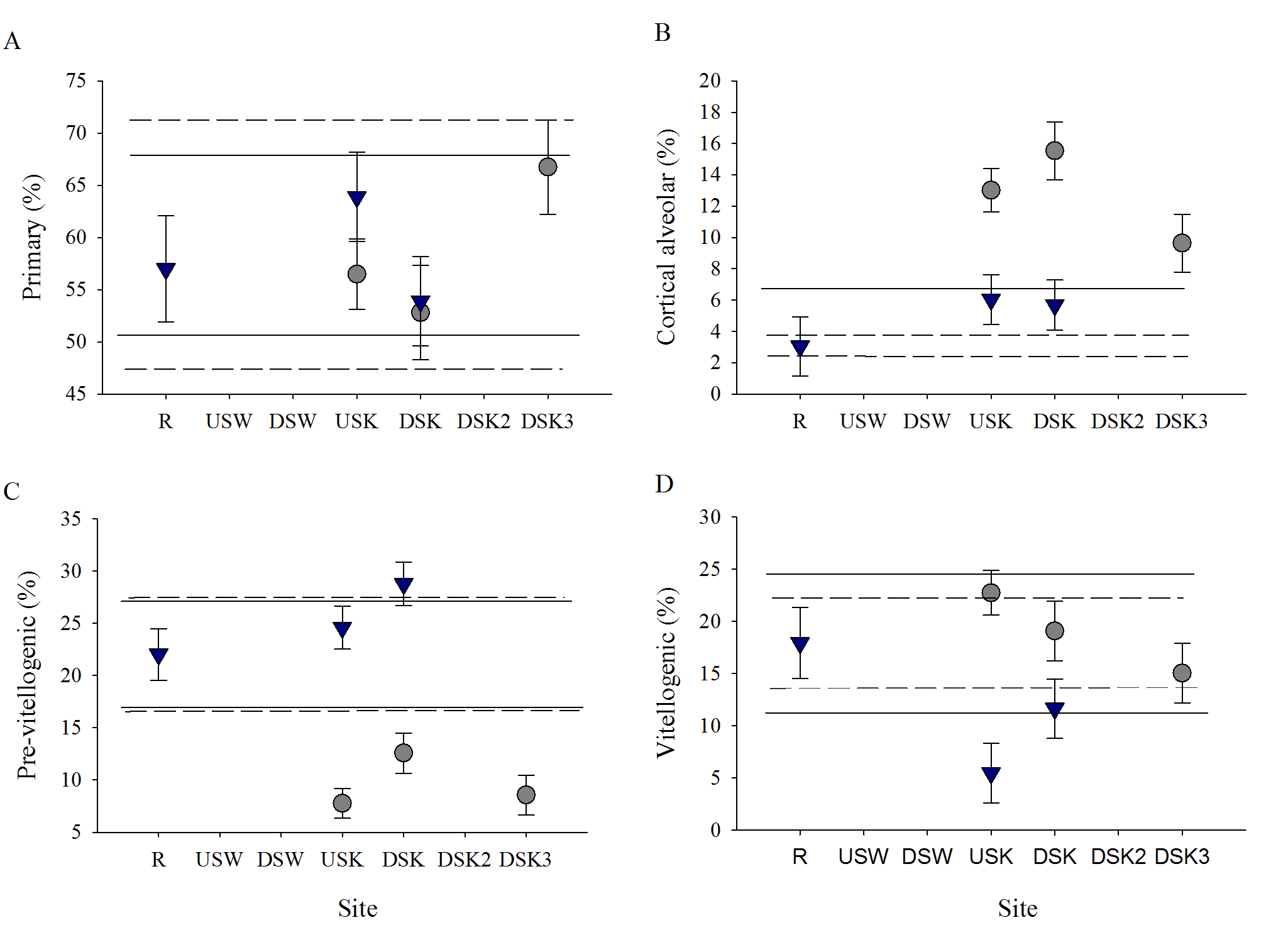

Supplement: S2 Fig — Relative proportion of primary (A), cortical alveolar (B), pre-vitellogenic (C), and vitellogenic (D) oocytes represented by the mean (± SE). Assessment was determined through histological analysis of female gonads collected in spring field season of 2009 (grey circles) and 2012 (blue down-facing triangles). The dashed line indicates the upper 25% critical effect size calculated from the mean of the data from the rural reference site (R), and the solid line indicates the upper 95% confidence interval from the pooled R. (TIF) [file pone.0164879.s003.TIF]

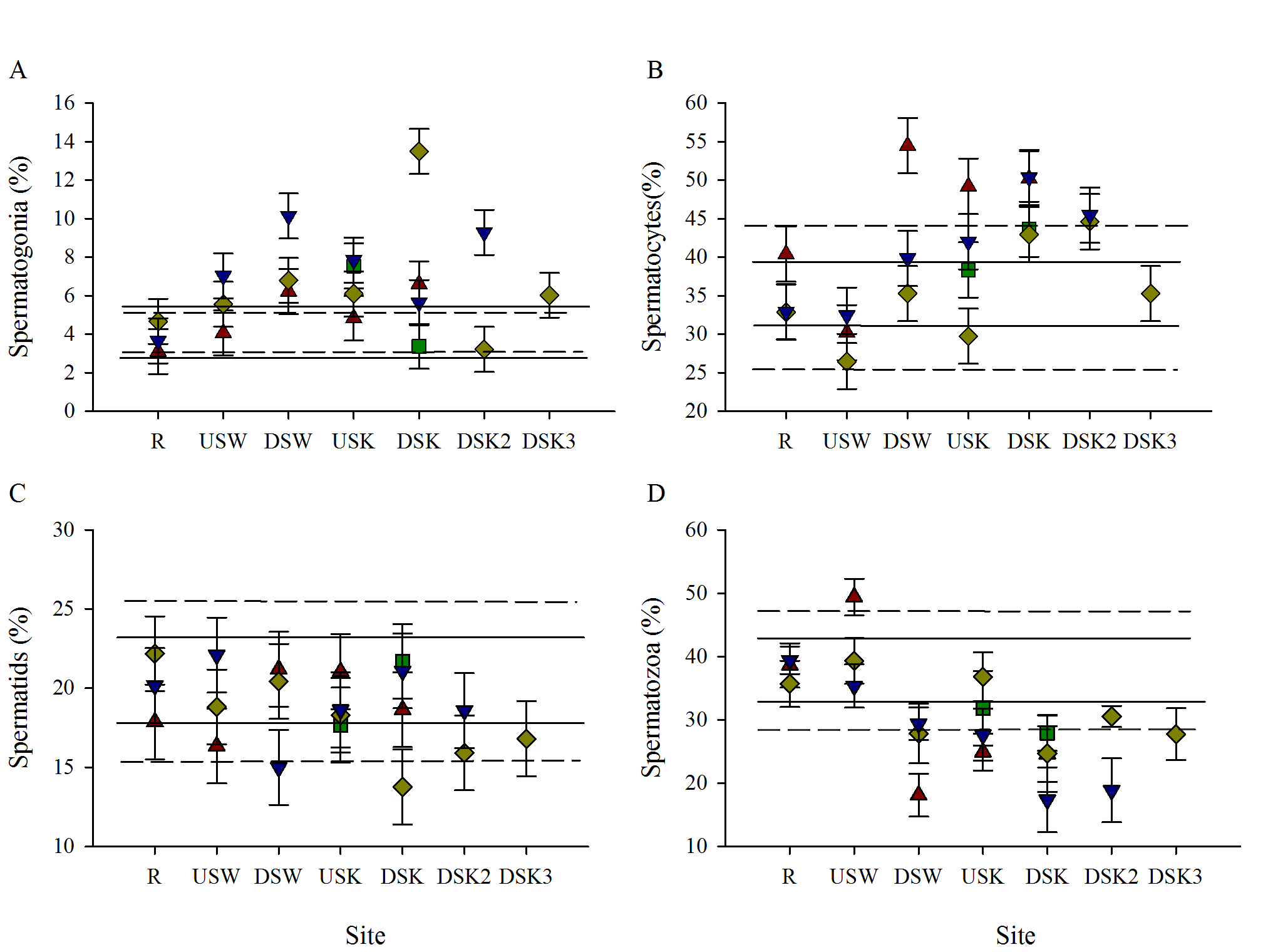

Supplement: S3 Fig — Relative proportion of spermatogonia (A), spermatocytes (B), spermatids (C), and spermatozoa (D) represented by the mean (± SE). Assessment was determined through histological analysis of male gonads collected in fall field season of 2007 (red triangles), 2010 (green squares), 2011 (yellow diamonds), and 2012 (blue down-facing triangles). The dashed line indicates the upper 25% critical effect size calculated from the mean of the data from the rural reference site (R), and the solid line indicates the upper 95% confidence interval from the pooled R. (TIF) [file pone.0164879.s004.TIF]

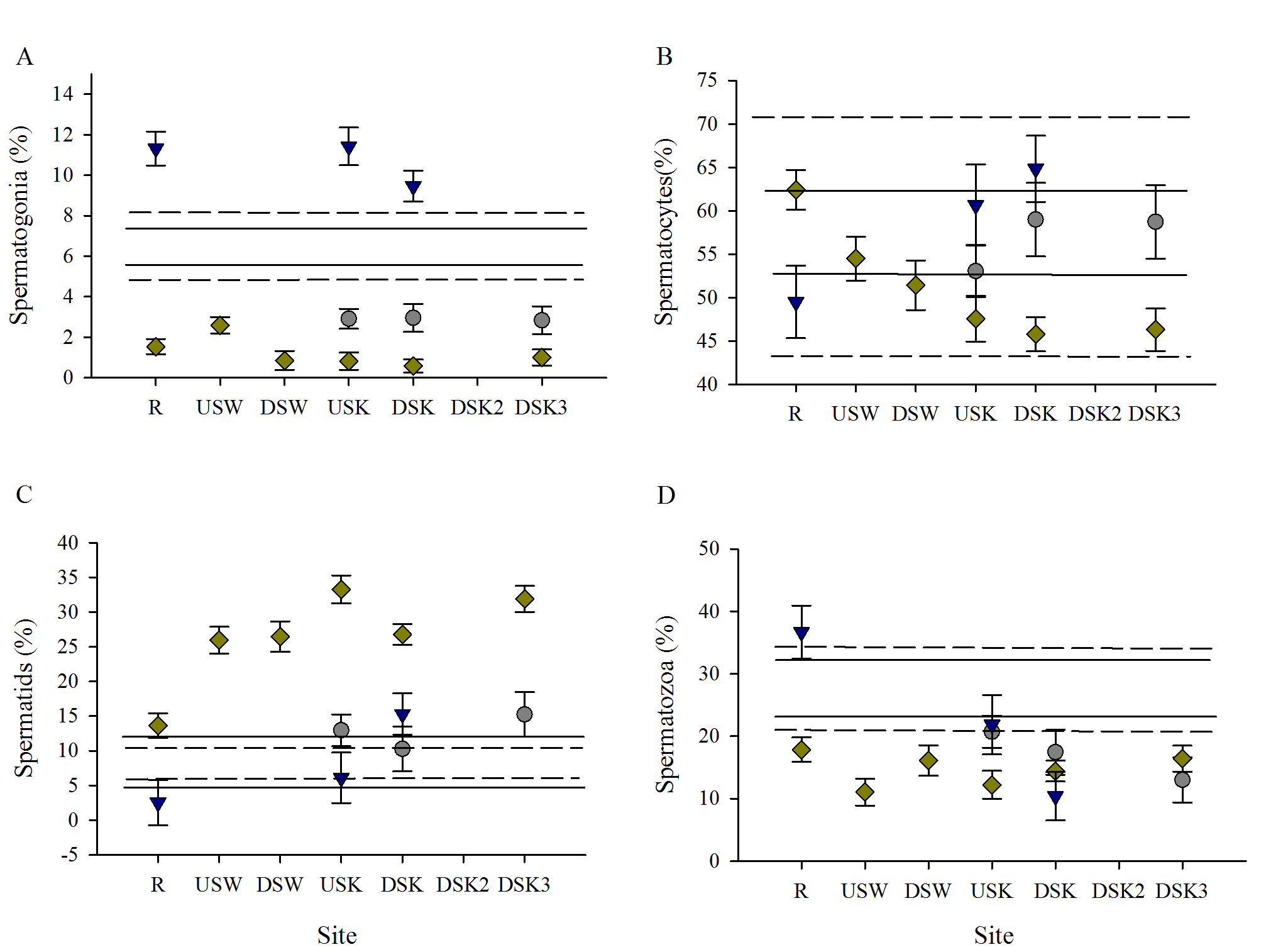

Supplement: S4 Fig — Relative proportion of spermatogonia (A), spermatocytes (B), spermatids (C), and spermatozoa (D) represented by the mean (± SE). Assessment was determined through histological analysis of male gonads collected in spring field season of 2009 (grey circles), 2011 (yellow diamonds), and 2012 (blue down-facing triangles). The dashed line indicates the upper 25% critical effect size calculated from the mean of the data from the rural reference site (R), and the solid line indicates the upper 95% confidence interval from the pooled R. (TIF) [file pone.0164879.s005.TIF]
